# Assessing the Effect of Screen Time on Physical Activity in Children Based on Parent-Reported Data: A Cross-Sectional Study

**DOI:** 10.7759/cureus.82971

**Published:** 2025-04-25

**Authors:** Malek Nasrallah, Amal Abu Helwa, Noora Y Jawhar, Almasah Alshammari, Amad R Jamal Eddin, Hasan BaniHani, Mode N Al Ojaimi

**Affiliations:** 1 Medicine and Surgery, University of Sharjah, Sharjah, ARE; 2 Pediatrics and Child Health, American University of Beirut, Beirut, LBN

**Keywords:** bmi, childhood obesity, pediatric public health, physical activity, screen exposure time, screen-time, sedentary lifestyle, united arab emirates (uae)

## Abstract

Introduction: The rise in screen time among children has raised concerns about its impact on physical activity and overall health. In the United Arab Emirates (UAE), rapid urbanization, high digital accessibility, and increasing childhood obesity rates necessitate further investigation into this relationship. This study aims to assess the impact of screen time on the physical activity and BMI of children aged 4 to 17 years in the UAE, using parent-reported data.

Methods: This was a cross-sectional study involving a target group of parents of children attending UAE schools. A self-administered questionnaire collected data on screen time, physical activity, eating habits, weight, and height of children. In this study, 'weekday' refers to school days, which vary by emirate. For instance, in Dubai, the school week runs from Monday to Friday, while in Sharjah, it runs from Monday to Thursday. Weekend screen time was not assessed in the study. BMI classifications followed CDC guidelines. Data from 300 participants were analyzed using SPSS, version 22 (IBM Corp., Armonk, NY), applying chi-square tests with a significance level of p < 0.05.

Results: A significant proportion (37.7%) of children spent more than seven hours per weekday on screens, with screen time increasing with age. High screen time was associated with lower physical activity levels, as 68.8% of children who exceeded seven hours of screen time did not participate in any physical activity. A strong correlation was observed between screen time and BMI, with higher screen exposure linked to overweight and obesity.

Conclusion: Excessive screen time negatively impacts children's physical activity and BMI, contributing to obesity concerns in the UAE. These findings highlight the need for targeted interventions involving parents, schools, and policymakers to reduce screen time and encourage active lifestyles.

## Introduction

The increasing prevalence of screen time among children has raised concerns about its impact on their physical activity and overall health. In an era where digital devices are widely accessible, children often spend long hours engaging with screens, leading to sedentary lifestyles. Numerous studies have explored the relationship between screen time, physical activity, and health outcomes, particularly obesity [1-3]. However, there is a need to further investigate this relationship within the context of the United Arab Emirates (UAE), where rapid urbanization, high accessibility to digital devices, and rising childhood obesity rates present unique challenges [4].

A study conducted in Japan found that increased screen time duration and timing were significantly associated with increased obesity, physical activity, and lower academic performance in elementary school children. The research revealed that prolonged screen time contributes to decreased physical activity and increased obesity, particularly when screen use occurs just before bedtime, further exacerbating the negative health outcomes [1]. Similar patterns have been observed globally. For instance, a study from Cyprus that included kids demonstrated that sedentary behaviors, such as watching TV for four or more hours per day, significantly increased the likelihood of obesity and abdominal obesity, particularly in girls. However, it is important to note that the study did not consider pubertal status or menarche, which may influence gender-based differences in obesity risk. Interestingly, this study found that sedentary behaviors were more important predictors of obesity than physical activity levels [2]. The COVID-19 pandemic exacerbated these concerns, as lockdowns led to a marked increase in screen time and sedentary behavior. Globally, nearly 37 million children under five are affected by obesity, with screen time and sedentary lifestyles identified as major contributors [5,6]. A comprehensive review of global studies in children have highlighted the link between increased screen usage, reduced physical activity, and the subsequent rise in childhood obesity and related health issues, including early markers of type 2 diabetes and cardiovascular risk [3]. While global studies have highlighted these trends, there is a lack of UAE-specific data assessing the combined influence of screen time and lifestyle factors on children’s health outcomes. This study provides a unique perspective by integrating data reported by parents in the UAE and evaluating secondary factors like eating habits and weight changes, addressing a significant gap in the literature.

This study seeks to assess the impact of screen time on the physical activity of children aged 4 to 17 years in the UAE, based on parent-reported data. By evaluating secondary factors such as eating habits and time spent on physical activity, this research aims to provide insights that can inform parents and policymakers about the potential risks associated with prolonged screen use. The approach of parent-reported data was chosen as the primary data collection method due to its practicality in assessing lifestyle factors in younger children, who may be less reliable when self-reporting their activities.

Our hypothesis is that children who spend more time on digital screens are less physically active and, consequently, more at risk of developing health issues such as obesity. Through this research, we hope to raise awareness about the importance of limiting screen time and encouraging more active lifestyles in children.

## Materials and methods

This was a cross-sectional study that explored the relationship between screen time and physical activity among children. This method was chosen because it allows us to measure both the outcome (physical activity) and the exposure (screen time), at the same time, making it a practical way to understand how these factors interact with the UAE’s population. Our target group was parents of children aged 4-17 years attending schools across the UAE between March and April, 2022. Children who were 4-17 years of age and were able to engage in physical activity were included. Children who were unable to engage in physical activity, did not have access to screens, or were studying online were excluded. The sampling method used was a volunteering approach where participants willingly joined the study, which allowed us to gather data from a broad and diverse group of individuals. To determine our sample size, we used a formula based on the expected prevalence, taking into account data from a similar study conducted in Turkey [7]. The calculation of the sample size led us to a minimum sample size of 215 participants, which we then increased by 10% to 237 to account for any potential missing data and reduce the risk of error.

The study was reviewed and approved by the research ethics committee of University of Sharjah (approval REC-22-02-16-11-S), ensuring that all ethical guidelines were adhered to throughout the research process. Informed consent was obtained from all participants prior to their enrollment in the study. We reassured all volunteers that their responses and all related research data would be treated with strict confidentiality, accessible only to the research team and our supervising doctor.

Data collection was carried out through a self-administered questionnaire that we developed specifically for this research. The questionnaire was pilot-tested for clarity, but no formal statistical validation (e.g., Cronbach’s alpha) was conducted. The questionnaire was distributed as an online form. A secure survey link was shared with participating schools, which then forwarded the link to enrolled parents who voluntarily completed it. The questionnaire included two main sections: one for demographic information and another for collecting data on screen time and physical activity, with a total of 13 questions. The questionnaire included questions about the type of screen, duration spent on screens per week, physical activity, its frequency and type, as well as snacking habits and the type of snacks consumed. The questionnaire also collected information on body weight and height, which were used to calculate the body mass index (BMI, kg/m²). For children and adolescents, BMI was classified using the CDC Child and Teen BMI categories [8]: Underweight, less than the 5th percentile; Healthy weight, 5th to less than 85th percentile; Overweight, 85th to less than 95th percentile; Obesity, 95th percentile or higher. We used simple language and provided an Arabic version of the questionnaire to make it more accessible to all participants (Table 1). The translation was done by a professional translator at the University of Sharjah, Professor Shehdeh Fareh at the College of Arts, Humanities, and Social Sciences. To ensure the questionnaire was clear and effective, we pilot-tested it with five parents, confirming that it took about five minutes to complete and was easy to understand by parents of all backgrounds. To approach our participants, we contacted schools to distribute the questionnaire to the parents (Table 1).

**Table 1:** Questionnaire AED: United Arab Emirates dirham

Questions	Options
Q1. What is your child’s gender?	Female
	Male
Q2. How old is your child?	____
Q3. What is your child’s height?	____
Q4. What is your child’s weight?	____
Q5. Which curriculum does your child’s school follow?	American
	Australian
	British
	Canadian
	French
	Indian
	Ministry
	Others: ____
Q6. What is your child’s school fees in AED?	<10,000
	10,000–49,999
	50,000–79,999
	>80,000
Q7. Which screen type does your child spend the most time on?	Phone
	iPad/Tablet
	TV
	Video games
	Laptop/computer
Q8 a. Most of your child’s leisure time is spent: (If your answer is “Not on screens”, skip to Q9 a)	On screens
	Not on screens
Q8 b. How long does your child spend on screens in general per weekday?	Less than 2 hours
	3 hours
	4-6 hours
	7+ hours
Q9 a. Is your child physically active? (If your answer is “No”, skip to Q10 a)	Yes
	No
Q9 b. How often is your child active (outside of school) during the week?	7 times a week
	3-5 times a week
	1-2 times a week
	0 times a week
Q9 c. What form of activity does your child perform? (You may choose more than one)	Home activities
	Leisure time activities
	Attends an organized sport (sports club, gymnastics, martial arts program)
Q10 a. Other than the daily meals, does your child eat any extra snacks while using a screen? (If your answer is "No", skip to Q11 a)	Yes
	No
Q10 b. What type of snacks does your child eat the most while using a screen?	Vegetables/fruits
	Sweets
	Soft drinks
	Fast food
Q11 a. Do you think screen time has affected your child’s physical activity?	Yes
	No
Q11 b. Do you think screen time has affected your child’s eating habits?	Yes
	No
Q12. What measures have you taken to limit screen time for your child? (open-ended question)	____

Contact numbers were provided to receive any inquiries from the participants. After collecting the data, we coded and analyzed it using SPSS, version 22 (IBM Corp., Armonk, NY). In total, data from 300 participants were included in the analysis. It included univariate statistics to summarize the data such as frequency, mean, and standard deviation along with bivariate analysis to examine the relationships between variables using the chi-square test. We set the significance level at 5% to ensure that our findings were statistically reliable. All reported p-values were two-sided and p-values < 0.05 were considered statistically significant.

## Results

The results of the study are presented in two sections: Demographics and Multivariate Analyses. These sections outline the key findings related to screen time, physical activity, BMI, and other associated factors among children in the UAE.

Demographics

The demographic analysis was conducted on a sample of children aged between 4 and 17 years, with a mean age of 9.71 years (SD = 3.424). The sample consisted of 59.3% male individuals and 40.7% female individuals. The height of participants ranged from 65 cm to 193 cm, with a mean of 138.58 cm (SD = 21.732), and weights ranged from 13 kg to 107 kg, with a mean of 38.86 kg (SD = 18.164). The mean BMI was calculated to be 19.37 kg/m² (SD = 5.028), with values ranging from 10.7 kg/m² to 38.8 kg/m².

Participants were selected from various educational curricula, including British (45.9%), Ministry (32%), American (15.5%), French (5.3%), Indian (0.3%), and Others (1%). The socioeconomic status, as inferred from school tuition fees, revealed that 8% of students paid less than 10,000 United Arab Emirates dirham (AED) per year, 82% paid between 10,000 and 49,999 AED annually, 9% paid between 50,000 and 79,999 AED per year, and 0.7% paid over 80,000 AED annually (Table 2).

**Table 2:** Demographics AED: United Arab Emirates dirham

Category		Value	Percentage
Gender	Male	178	59.3%
	Female	122	40.7%
Age	Mean	9.71	-
	Standard deviation	3.424	-
	Min	4	-
	Max	17	-
Height (cm)	Mean	138.58	-
	Standard deviation	21.732	-
	Min	65	-
	Max	193	-
Weight (kg)	Mean	38.86	-
	Standard deviation	18.164	-
	Min	13	-
	Max	107	-
BMI (kg/m^2^)	Mean	19.37	-
	Standard deviation	5.028	-
	Min	10.7	-
	Max	38.8	-
Curriculum	American	47	15.7%
	British	136	45.3%
	French	16	5.3%
	Indian	1	0.3%
	Ministry	97	32.3%
	Other	3	1.0%
School fees (AED)	<10,000	25	8.3%
	10,000-49,999	246	82.0%
	50,000-79,999	27	9.0%
	>80,000	2	0.7%

Multivariate analyses

Screen Time and Age

It was found that 37.7% of children spent more than seven hours per weekday on screens, with screen time increasing significantly with age. For children aged 7 years or younger, 32.1% spent four to six hours on screens per weekday, while for those aged 12 years and older, 50% spent seven or more hours on screens (p = 0.047) (Table 3).

**Table 3:** Screen time and age

Screen time	Age group			
	≤7	8-11	≥12	Total
<2 hours	17.9%	8.1%	9.3%	11.3%
3 hours	23.2%	22.6%	14.0%	19.1%
4-6 hours	32.1%	38.7%	26.7%	31.9%
≥7 hours	26.8%	30.6%	50.0%	37.7%

Screen Time and Physical Activity

Children who spent most of their leisure time on screens were less likely to engage in physical activity; 45.1% of these children did not participate in any physical activity compared to only 10.4% of those whose leisure time was not screen-dominated (p = 0.001). Additionally, 68.8% of children who spent more than seven hours on screens per weekday did not participate in physical activity, whereas 87.0% of those spending less than two hours were active (p = 0.001) (Table 4).

**Table 4:** Screen time and physical activity

Physical activity	Screen time				
	<2 hours	3 hours	4-6 hours	≥7 hours	Total
Yes	87.0%	71.8%	61.5%	31.2%	54.9%
No	13.0%	28.2%	38.5%	68.8%	45.1%

BMI and Screen Time

A strong correlation was observed between screen time and BMI. Among children who spent less than two hours on screens per weekday, 73.9% were of healthy weight, while 46.8% of those who spent seven or more hours were of healthy weight. Notably, 22.1% of children with seven or more hours of screen time were found to be overweight, compared to only 8.7% among those with less screen exposure (p < 0.05) (Table 5).

**Table 5:** BMI and screen time

BMI category	Screen time			
	<2 hours	3 hours	4-6 hours	≥7 hours
Underweight	8.7%	5.1%	9.2%	5.2%
Healthy weight	73.9%	56.4%	44.6%	46.8%
Overweight	8.7%`	10.3%	26.2%	22.1%
Obese	8.7%	23.1%	15.4%	22.1%

BMI and Physical Activity

Analysis showed that physical activity was positively associated with a healthy BMI. Among children who participated in physical activity, 53.5% were of healthy weight, compared to 40.2% among those who did not participate in any physical activity. Conversely, higher proportions of overweight (27.5%) and obese (20.6%) children were observed among those who were inactive (p < 0.05) (Table 6).

**Table 6:** BMI and physical activity

BMI category	Physical activity	
	Yes	No
Underweight	12.6%	18.7%
Healthy weight	53.5%	40.2%
Overweight	12.6%	27.5%
Obese	18.7%	20.6%

Parental Measures and BMI

Parental measures to limit screen time varied, including setting schedules, encouraging alternative activities, and using rewards or punishments. The most effective method was found to be guiding the child, as 66.7% of children whose parents used guidance were of healthy weight. In contrast, 24.1% of children whose parents took no measures to limit screen time were overweight, highlighting the critical role of parental involvement (p < 0.05) (Table 7).

**Table 7:** Parental measures and BMI

BMI category	Measures taken					
	Activities	Scheduling/limitations	No measures	Reward and punishment	Guiding	Unanswered
Underweight	17.5%	7.3%	6.9%	16.7%	0.0%	8.6%
Healthy weight	19.0%	17.1%	17.2%	33.3%	11.1%	24.3%
Overweight	41.3%	53.7%	48.3%	50.0%	66.7%	45.7%
Obese	19.0%	17.1%	24.1%	0.0%	22.7%	15.7%
Left blank	3.2%	4.9%	3.4%	0.0%	0.0%	5.7%

Gender and Physical Activity

There were no significant gender differences in physical activity levels; 68.5% of boys and 62.3% of girls were physically active. However, slight differences in screen time usage were noted, with male individuals tending to spend more time on screens than female individuals (p < 0.262) (Table 8).

**Table 8:** Gender and physical activity

Gender	Physical activity	
	Yes	No
Male	68.5%	31.5%
Female	62.3%	37.7%

Gender and Screen Time

The relationship between gender and screen time was examined, with a comparison made on the amount of time boys and girls spent on screens during weekdays. The results showed a slight difference in screen time habits between boys and girls. A larger percentage of boys (40.2%) spent seven or more hours per weekday on screens, while 30.5% of boys spent four to six hours per weekday on screens. On the other hand, a lower percentage (32.4%) of girls spent seven or more hours on screens, while 34.2% of girls spent four to six hours on screens. These findings suggest that boys generally tend to spend more time on screens compared to girls. This difference may reflect gender-related preferences in activities, such as boys engaging more in video games and other digital media during their leisure time. The analysis also showed that a higher proportion of girls (16.5%) spend less than two hours per weekday on screens, compared to only 9.3% of boys.

Curriculum and Physical Activity

Physical activity levels varied across educational curricula. The highest activity levels were reported among children in the French curriculum (81.3%), while the Ministry curriculum had the lowest activity levels (54.6%) (p < 0.015) (Table 9).

**Table 9:** Curriculum and physical activity

Physical activity	Curriculum				
	Ministry	American	British	French	Others
Yes	54.6%	59.6%	74.3%	81.3%	75.0%
No	45.4%	40.4%	25.7%	18.8%	25.0%

Curriculum and BMI

The bivariate analysis comparing BMI and curriculum revealed distinct patterns across educational environments. The French curriculum had the highest percentage of children with healthy weight (75.0%) and the lowest percentage of underweight (6.3%) and obese children (0%), suggesting more favorable health outcomes. In contrast, the British curriculum had the highest proportion of obese children (24.3%) and underweight children (12.5%). Similarly, the American curriculum showed high rates for both obesity (21.3%) and underweight children (19.1%). The Ministry curriculum had the highest percentage of overweight children (47.2%), but a relatively lower percentage of obesity (14.4%) compared to British and American systems (p < 0.027) (Table 10).

**Table 10:** Curriculum and BMI

BMI category	Curriculum				
	American	British	French	Ministry	Others
Underweight	19.1%	12.5%	6.3%	2.1%	0.0%
Healthy weight	40.4%	44.9%	75.0%	54.6%	50%
Overweight	14.9%	13.2%	12.5%	25.8%	25.0%
Obese	21.3%	24.3%	0.0%	14.4%	25.0%

## Discussion

The present study aimed to investigate the relationship between screen time and its effects on physical activity, BMI, and dietary habits among children in the UAE. By collecting data from a diverse sample of children and analyzing it through various demographic lenses, we uncovered several significant findings that have important implications for public health and education policies in the UAE, particularly in understanding how screen time, physical activity, and BMI are interconnected. This study also explored the influence of different curricula on these health outcomes, shedding light on the role of educational environments in shaping children's lifestyles.

Our study revealed that a substantial proportion of children (68%) spent most of their leisure time on screens. This high prevalence of screen use is concerning, given its association with physical inactivity and increased BMI. Specifically, we found that 37.7% of the children used their screens for more than seven hours on weekdays, which is significantly higher than the recommended two hours per day [9]. The excessive screen time observed may explain why many of the children are not engaging in physical activity required to maintain a healthy weight. When examining physical activity levels, we found that although a majority of children (66%) were reported to be physically active, a notable 34% were not. This discrepancy highlights the negative impact of excessive screen time on physical activity, as children who spend more time on screens are less likely to engage in physical activities. This pattern aligns with the well-documented relationship between sedentary behavior and a higher risk of weight gain, which was evident in our findings [10]. Our BMI analysis showed a mean BMI of 19.37 kg/m², with a wide range from 10.7 to 38.8 kg/m². The data indicated that a significant portion of the sample had BMIs above the healthy range, which is consistent with the high levels of screen time and physical inactivity observed. These findings echo previous research suggesting that increased screen time is a risk factor for childhood obesity [1,2], as it displaces time that could be spent on more physically engaging activities [11].

A distinctive feature of this study was its focus on examining the impact of different school curricula on children’s health behaviors and outcomes. The results indicated significant differences in BMI and physical activity levels based on the curriculum the children followed. The high percentage of overweight and obese children in the Ministry curriculum is a particularly notable finding. Several factors could contribute to this outcome. Firstly, the Ministry curriculum predominantly serves Arab nationals, a population that has historically faced increasing rates of obesity [12]. Cultural factors such as dietary preferences, which include foods high in sugar and fat, combined with a more sedentary lifestyle, could contribute to the higher BMI observed in these children [13].

As the country has become more developed, there has been an increase in sedentary lifestyles, with fewer children participating in outdoor activities or sports. Social and environmental factors, such as reliance on cars for transportation, urbanization, and the availability of convenient yet unhealthy food options exacerbate these issues [14]. The hot climate in the UAE serves as a significant barrier to physical activity, particularly during the long summer months when outdoor temperatures can become unbearable. This extreme heat discourages outdoor exercise and limits opportunities for physical activity, especially in urban areas where shaded or climate-controlled spaces may be scarce [15].

The implications of our findings are multifaceted and underscore the need for targeted interventions to reduce screen time and promote physical activity, particularly among UAE nationals and students in the Ministry curriculum. Schools and parents play a crucial role in addressing this issue. The UAE Ministry of Health and Prevention (MoHAP) conducted a workshop to develop an updated National Programme to Combat Obesity in Children and Adolescents, aligning with the UAE's sustainability and health priorities for the next 50 years. The plan aims to promote healthy lifestyles, prevent obesity-related diseases, and improve children's quality of life, aligning with WHO’s action plan on non-communicable diseases. Key initiatives include a multidisciplinary approach addressing obesity at early stages, integrating interventions, educational campaigns, and collaboration with government and private sectors. Additionally, the 'Mutabah' system, launched in 2020, monitors obesity and offers tailored guidance on diet and physical activity for students [16]. MoHAP launched a back-to-school health awareness campaign from August 17 to 31, 2023, focusing on promoting healthy lifestyles among students and parents. The initiative included community events, cooking workshops, nutrition advice, and discussions with health educators. It also emphasized vaccination and raised awareness about seasonal influenza and respiratory illnesses. Aligned with the WHO's "Health-Promoting Schools" initiative, the campaign sought to create a safe, health-conscious school environment, encouraging balanced nutrition, physical activity, and overall student well-being [17]​.

Given these initiatives, it is essential to integrate findings from our study into ongoing efforts. Targeted interventions that reduce sedentary behavior and increase physical activity should be prioritized, with a focus on children and adolescents. Additionally, fostering collaboration among schools, parents, and health authorities is critical to sustaining long-term positive changes in student health behaviors. Parents should be encouraged to set clear limits on screen time and promote alternative activities, such as sports, outdoor play, and family outings. In addition, addressing dietary habits is crucial. With 62.1% of children consuming sweets while using screens, there is a clear need for nutritional education and healthier snack options. Schools can implement programs to educate children about balanced diets and the importance of healthy eating alongside promoting physical activity.

Limitations

Despite the valuable insights provided, this study has several limitations. The reliance on self-reported data from parents introduces a potential bias, as parents may underreport or overreport their child’s screen time and physical activity. Heights and weights of the children were also measured by parents, which may be inaccurate and subject to reporting errors. One extreme value in the dataset - a reported height of 65 cm - was identified and is likely due to a parent-reported data entry error. Although such outliers were rare, they may affect the accuracy of BMI calculations for individual cases. However, given the categorical analysis of BMI and the sample size, this anomaly is unlikely to have impacted overall study trends. This reflects a broader limitation of self-reported anthropometric data, which may contain occasional inaccuracies. Furthermore, the questionnaire was not validated using standard statistical methods such as internal consistency. Additionally, the questionnaire did not provide a standardized definition of “physical activity,” which may have led to varying interpretations among parents when answering related items. The cross-sectional design of the study does not allow for causal inferences, making it difficult to determine whether screen time leads to physical inactivity or vice versa. Longitudinal studies are needed to establish causality between screen time, physical activity, and BMI.

Moreover, the study sample may not be fully representative of the entire UAE population, given the higher response rates from British and Ministry curricula. Additionally, the research did not collect data on participants’ ethnicities, which may influence lifestyle patterns. The study also did not assess screen time during weekends, which could vary across emirates due to differing school week structures; for example, schools in Dubai operate from Monday to Friday, while those in Sharjah follow a Monday to Thursday schedule. Future research should aim to include a more balanced representation of various educational systems, public schools, and socioeconomic backgrounds to provide a more comprehensive understanding of these issues.

Future directions

Future research should explore the longitudinal effects of screen time on children's health outcomes to establish causality. Additionally, intervention studies that test the effectiveness of various strategies to reduce screen time and promote physical activity are needed, especially in Ministry schools. These studies could provide valuable insights into the best practices for improving children's health in the UAE, particularly among UAE nationals who face higher risks of obesity and related health issues.

## Conclusions

In conclusion, our study provides compelling evidence that children who maintain a healthy weight and spend less time on screens are more likely to be physically active. Excessive screen time is strongly associated with reduced physical activity and higher BMI, which can lead to various health conditions. These findings highlight the need for families to take proactive measures to limit screen time and encourage more physical activity in their children. The study’s findings could contribute to the development of public health strategies aimed at combating childhood obesity and promoting healthier lifestyles in the UAE.
